# Comparison of Ultra-Conserved Elements in Drosophilids and Vertebrates

**DOI:** 10.1371/journal.pone.0082362

**Published:** 2013-12-13

**Authors:** Igor V. Makunin, Viktor V. Shloma, Stuart J. Stephen, Michael Pheasant, Stepan N. Belyakin

**Affiliations:** 1 Research Computing Centre, The University of Queensland, Brisbane, Queensland, Australia; 2 Institute of Molecular and Cellular Biology SD RAS, Novosibirsk, Russia; 3 Computational Biology Group, CSIRO Plant Industry, Canberra, Australian Capital Territory, Australia; Inserm U869, France

## Abstract

Metazoan genomes contain many ultra-conserved elements (UCEs), long sequences identical between distant species. In this study we identified UCEs in drosophilid and vertebrate species with a similar level of phylogenetic divergence measured at protein-coding regions, and demonstrated that both the length and number of UCEs are larger in vertebrates. The proportion of non-exonic UCEs declines in distant drosophilids whilst an opposite trend was observed in vertebrates. We generated a set of 2,126 Sophophora UCEs by merging elements identified in several drosophila species and compared these to the eutherian UCEs identified in placental mammals. In contrast to vertebrates, the Sophophora UCEs are depleted around transcription start sites. Analysis of 52,954 *P-element*, *piggyBac* and *Minos* insertions in the *D. melanogaster* genome revealed depletion of the *P-element* and *piggyBac* insertions in and around the Sophophora UCEs. We examined eleven fly strains with transposon insertions into the intergenic UCEs and identified associated phenotypes in five strains. Four insertions behave as recessive lethals, and in one case we observed a suppression of the marker gene within the transgene, presumably by silenced chromatin around the integration site. To confirm the lethality is caused by integration of transposons we performed a phenotype rescue experiment for two stocks and demonstrated that the excision of the transposons from the intergenic UCEs restores viability. Sequencing of DNA after the transposon excision in one fly strain with the restored viability revealed a 47 bp insertion at the original transposon integration site suggesting that the nature of the mutation is important for the appearance of the phenotype. Our results suggest that the UCEs in flies and vertebrates have both common and distinct features, and demonstrate that a significant proportion of intergenic drosophila UCEs are sensitive to disruption.

## Introduction

Comparative analysis of mammalian and insect genomes have demonstrated that the majority of the evolutionarily constrained sequences in these lineages are located outside of protein coding regions [1], [2]. Comparison of the human and mouse sequences on chromosome 21 showed that many non-coding sequences are even more conserved than protein-coding regions [3]. Subsequent studies then identified numerous highly conserved non-coding elements (CNEs) in species as evolutionarily distant as human and fish [4], [5], which are clustered around genes involved in regulation of transcription and development [6].

The Ultra-Conserved Elements (UCEs) are arguably the most constrained sequences in the human genome. The UCEs were first identified as sequences at least 200 bp long identical between the human, mouse and rat genomes [7]. Another study described 13,736 human UCEs identical over at least 100 bp in at least 3 of 5 placental mammals [8], and shorter UCEs were identified between human and phylogenetically distant species such as sponge, sea anemone, fruit fly and sea urchin [9]. An alignment-independent method was used for the identification of both syntenic and non-syntenic Long Identical Multispecies Elements in vertebrates and plants [10] including some elements omitted in earlier studies due to gap alignment deficiencies.

While UCEs were identified on the basis of sequence identity, they likely fall into several functional categories. The majority of UCEs do not overlap with protein-coding regions. Some exonic UCEs are apparently involved in regulation of splicing [11], [12]. Non-exonic UCEs can act as enhancers [13], [14]. Many UCEs are transcribed [15]–[17]. It is still unknown why UCEs maintain 100% sequence conservation over such long stretches of DNA, considering that the majority of known protein-binding sites are relatively very short. One possible explanation would be an overlap of several constraints, for example enhancer/protein binding and non-coding RNA [18] or enhancer and protein-coding regions [19]. It seems that distances between the UCEs are also conserved [20] raising the possibility that such sequences could be involved in the maintenance of higher-order genome structure. To some extent the conservation of distances between the UCEs can be explained by the absence of annotated transposons in the vicinity of many UCEs [21]. Such transposon-free regions are maintained in bony vertebrates [22] and often coincide with so-called chromatin bivalent domains [23] hinting at a possible link between UCEs and chromatin. CNEs are apparently linked to maintenance of long syntenic regions in both vertebrates and insects [6], [24].

The observed extreme conservation within UCEs suggests a strong negative selection pressure, implying that any disruption of the UCEs may have dramatic negative consequences for an organism. Indeed, some mutations outside of protein-coding regions are very harmful [25]. However, deletion of four individual UCEs had no visible effect on mice [26]. Similarly, deletion of long “gene desert” regions containing many CNEs also had no detectible effect on mice viability or phenotype [27]. On the other hand, over evolutionary history, deletions of ultraconserved-like elements are observed to be over 300-fold less likely than deletions of neutral DNA [28]. Insertion of 16 bp sequences into the highly conserved Dc2 enhancer of *DACH1* gene did not cause any detectable changes in expression of a marker gene [29]. One study described an association between SNPs in UCEs and breast cancer [30], but the results were not reproduced in a different population [31]. While some UCEs are altered in cancer [15] it is not clear whether such changes represent driver or passenger mutations. SNPs in UCEs associate with complex traits such as BMI and height but show no transmission bias from parents to children, ruling out any strongly deleterious effect of these rare alleles [32].

It is plausible that the disruption or deletion of UCEs may have a strong deleterious impact on survival in complex natural environments but would have very little effect on fitness under controlled lab conditions as observed for non-coding RNA BC1 [33]. Indeed, an SNP in an ultraconserved regulatory sequence is linked with *Dlx5*/*Dlx6* expression in the forebrain [34]. Another paper reported an enrichment of UCEs in chromosomal rearrangements, especially pathogenic deletions, identified in 200 people with idiopathic neurodevelopmental disorders [35].

Despite the extreme conservation in mammals, none of the non-coding human UCEs were traced outside the vertebrate lineage [7] even though thousands of human protein-coding genes have detectable orthologs in insects [36]. None of the 481 UCEs identified by Bejerano and co-authors [7] overlap with the UCEs identified between phylogenetically distant species such as human and demosponge, hydra or sea anemone [9]. However, a recent study has described 183 CNEs conserved between mammals, fishes and tunicates, of which 145 overlap with an extended set of 5,404 vertebrate UCEs [37]. This contradiction between the extremely high conservation of UCEs in mammals and amniotes and the nearly complete lack of homologs even in distant Chordata species could be explained by a non-uniform substitution rate in such sequences over evolutionary time. Analysis of the extended set of 13,736 UCEs revealed an extremely low substitution rate within amniotes whilst in amphibian and bony fish lineages these regions evolved with higher substitution rates [8]. Another study found that many UCEs identified in Eutherian species can be found in a cartilaginous fish, the elephant shark, but it also confirmed that a significant number of non-exonic UCEs had first appeared in tetrapods and amniotes [38].

It seems the changes in substitution rates within CNEs has been very common over evolutionary history [39]. Non-uniform evolutionary substitution rates can reflect changes of function by a sequence, either gaining of a new biological functionality or loss of the existing. Such change in function may transform selection pressure on the sequence. The term “exaptation” was coined to describe acquisition, or “cooption” of a new function with a positive effect on fitness [40]. Consistent with this idea, some UCEs originated from ancient mobile elements [41], [42] and their current sequence constraints are presumably no longer due to their original mobile element functionality. It is interesting to note that while there is no similarity between CNEs in distant species, some orthologous genes acquired highly conserved CNEs in different lineages: out of 156 human CNE-associated genes with invertebrate orthologs, 40 are also associated with CNEs in worms and flies [43].

UCEs have also been identified in insects [44], [45]. Accepting that a very limited number of insect species genome assemblies have been analyzed, the UCEs in these insects show somewhat different properties compared to vertebrates. They are less frequently associated with genes encoding transcriptional factors, the UCE elements are shorter and the longest UCEs tend to overlap splice sites or reside in exons [44]. The longest identified drosophila UCE, at an exon-intron junction of the *homothorax* (*hth*) gene, has a complementary sequence located downstream in an intron of the gene and potentially may form an alternative RNA secondary structure and regulate its alternative splicing [46].

In this work we compare UCEs in insects and vertebrates. Using available genome sequences from several Drosophila and vertebrate species we have identified and compared UCEs in insect and vertebrate species with similar phylogenetic distances estimated from protein-coding sequences. We analyzed transposon insertions within eleven intergenic UCEs and identified visible phenotypes in five fly strains including four lethals. To prove the link between transposon insertion into intergenic UCEs and lethality we performed “phenotype rescue” experiments for two fly stocks with *P-elements* insertions. The removal of the transposons from the intergenic UCEs restored viability, confirming the association between the insertions and lethality.

## Materials and Methods

### Sequences

The 15-way drosophila dm3 centric and the 28-way human hg18 centric alignment were downloaded from the UCSC Genome Browser website [47] and used as the reference datasets when identifying putative UCEs. A sliding window of 100 bp over the multiZ alignments was used to identify minimal length seeds which were 100% identical between the relevant subset of aligned species (*e.g.*, *D. melanogaster vs D. yakuba vs D. erecta*). Identified minimal length seeds were then maximally extended until either a base mismatch or the extent of the multiZ alignment region containing the species subset was reached. The following genome assemblies were used: dm3, droEre2, dp4, droAna4, droGri2, droMoj3, droVir3, droWil1, droYak2, hg18, canFam2, anoCar1, bosTau3, danRer4, fr2, galGal3, gasAcu1, mm8, monDom4, ornAna1, oryLat1, tetNig1, xenTro2. The vertebrate UCEs coordinates were converted to the hg19 by liftOver [48]. An UCE was considered conserved if at least 20 nucleotides were aligned in other species. We used FlyBase 5.12 and the refSeq genes annotations (dm3, July 30, 2012; hg19, August 1, 2012). A UCE with any overlap with an annotated exon was considered exonic.

### The Phylogenetic Distances for Insects and Vertebrates

The dm3 and hg18 genome annotations on the UCSC Genome browser [47] were queried with “rpl” and “rps” to identify ribosomal genes and the refSeq IDs were extracted. For the non-conserved gene set we arbitrary selected 306 Drosophila genes on chromosome 2L without annotated alignments to the *Anopheles gambiae* genome. Only one protein-coding isoform per refSeq gene was used (Dataset S1). The bed files for selected genes were extracted from the UCSC Table Browser and were uploaded to the Galaxy web site [49]. The alignments of coding regions were extracted using Stitch Gene blocks on 28-way multiZ alignment of hg18 or 15-way dm3 alignment and concatenated using the “Concatenate FASTA alignment by species” function. The concatenated alignments were imported into MEGA4 phylogenetic software [50]. Four-fold degenerate sites were exported as separate alignments and used for reconstruction of the phylogenetic trees in the PhyML software [51] with default parameters except that the ratio of transversions and transitions were auto-calculated. The pairwise distances for non-synonymous substitutions within ribosomal genes were calculated in MEGA4 with the Pamilo-Bianchi-Li model and the complete deletion option for gaps and missing data [50].

### Syntenic Blocks

We used the 22 largest syntenic blocks (HCBs) identified with conserved gene order (GO) criterion [52] and containing at least 21 independent gene anchors. The coordinates were converted to dm3 using the liftOver function. For the Chi-square test we assumed a uniform distribution of UCEs.

### Statistics

The expected proportion of insertions or TSS features in any region of interest was calculated as the proportion of that region's total length to the length of the genome (*i.e.*, assuming a random distribution of features across the genome). For UCEs outside of intercalary heterochromatin regions we used TSS and insertions located outside of those regions. Chi-square tests were calculated in Excel, and for 2×2 contingency tables we used an online calculator: http://faculty.vassar.edu/lowry/tab2×2.html. Gene Ontology analysis was performed using FuncAssociate 2.0 [53] at http://llama.med.harvard.edu/funcassociate/.

### Mutations/insertions in UCEs

Coordinates of insertion sites were downloaded from FlyBase [54] as following: the Insertions section of FlyBase was queried with “P{*”, “pBac{*” and “Mi{*” for *P-element*, *piggyBac* and *Minos* inserts, respectively. The data on the integration sites were downloaded using HitList Conversion tools. Data for the *P-elements* were downloaded on February 11, 2010, and *piggyBac*s and *Minos* on January 5, 2012. In total we used 33,481 *P-elements*, 15,355 *piggyBac* and 4,118 *Minos* inserts mapped with precision of less than 10 bp within euchromatin. Analysis of insertion and UCEs distribution was done using the UCSC Table Browser [47].

### Genetics and Molecular Biology

Flies were raised on a standard drosophila cornmeal–yeast– agar medium at 25°C. The fly stocks carrying insertions within intergenic Sophophora UCEs were ordered from Exelixis: *PBac{WH}f06142* (stock ID: f06142), *PBac{PB}c00059* (c00059), *PBac{WH}f02223* (f02223), *PBac{WH}f05912* (f05912), *P{XP}d07857* (d07857), *PBac{WH}f07151* (f07151), *PBac{WH}f02632* (f02632) and *PBac{PB}c06670* (c06670) [55]. Three other fly stocks were ordered from the Bloomington stock center: *P{SUPor-P}KG10325* (stock ID 15254), *PBac{WH}Rdl^f02994^* (18606), *P{SUPor-P}KG02042* (14258) [55], [56]. The *y w; Ki, P{ry^+^, Δ2–3}99B* (y[1] w[1]; Ki[1] P{ry[+t7.2] = Delta2–3}99B) flies used as source of the transposase (Bloomington stock center, http://flybase.org/reports/FBst0004368.html). The *y, w; If/CyO; MKRS/TM6b* stock was used for balancing chromosomes after *P-element* excisions. DNA around insertion site after excision of the *P-element* in *d07857* flies was amplified and sequenced with the following primers: d07857_1_d CACCCCTCCACCTAACCAA and d07857_1_r CGATCTGTGATCTTGTGATTGATC. The sequences were aligned to the *D. melanogaster* genome by BLAST at the FlyBase site [54].

## Results

### Comparison of Phylogenetic Distances in Vertebrates and Flies

The rate of substitution is higher in insect than in vertebrate genomes. For example, the *Sophophora* and *Drosophila* subgenera diverged ∼36 million years ago [57] and the Amniota diverged ∼310 million years ago, yet the phylogenetic distance (estimated from fourfold degenerate sites) between *D. melanogaster* (*Sophophora* subgenus) and *D. virilis* (*Drosophila* subgenus) exceeds the phylogenetic distance between the amniotes human and chicken [58]. In our work we focus on the comparison of UCEs in sets of species with similar phylogenetic distances.

**Figure 1:**
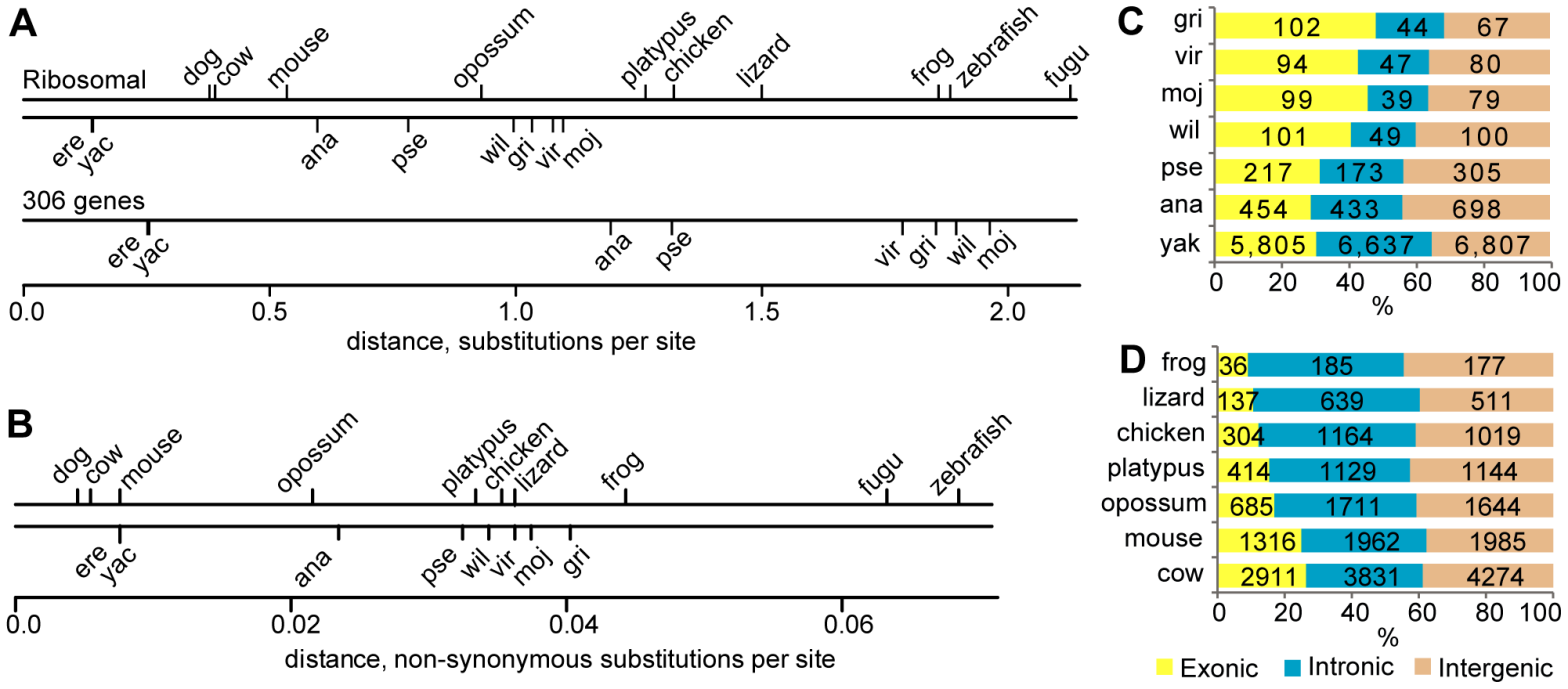
Comparison of phylogenetic distances and UCEs in drosophilids and vertebrates. (**A**) The pairwise distances at four-fold degenerate sites were estimated from the phylogenetic trees generated by PhyML package. The human and *D. melanogaster* genomes were used as a master sequence. Note the difference in distance estimations between the highly expressed genes encoding ribosomal proteins and 306 non-conserved genes in flies. Abbreviation for the Drosophila species: ere – *D. erecta*, yac – *D. yakuba*, ana – *D. ananassae*, pse – *D. pseudoobscura*, wil - *D. willistoni*, vir – *D. virilis*, moj – *D. mojavensis*, gri – *D. grimshawi*. (**B**) The pairwise distances for non-synonymous sites within the ribosomal genes. Only one transcript isoform per gene was used. (**C**) UCEs identified in different drosophila sets split into exonic, intronic and intergenic according to the refSeq genes (September 16, 2013). (**D**) UCEs identified in vertebrates. The fish sets were excluded due to a small number of the elements.

Phylogenetic distance can be measured by the neutral substitution rate, commonly estimated using divergence in assumed non-functional regions of the genome such as four-fold degenerate (synonymous) sites or syntenic ancient transposons, but this can be problematic comparing highly divergent genomes where sites become saturated with substitutions (*e.g.*, when the number of substitutions per site exceeds 1). Alternatively, relative phylogenetic distance can be estimated using non-synonymous substitutions in conserved protein-coding genes. Both approaches have problems. Drosophilids have very few transposons in the euchromatic part of their genomes; hence ancient transposons essentially cannot be used for estimation of the neutral substitution rate in flies. Divergence at four-fold degenerate sites has been used for comparative analysis of vertebrates and flies [58]. However, a very strong codon bias linked to gene expression level [59] results in a significant variation of the substitution rate at these sites in Drosophila genomes. To demonstrate the significance of this phenomena, we calculated the phylogenetic distances in four-fold degenerate sites of highly expressed genes encoding ribosomal proteins and 306 genes from *D. melanogaster* chromosome 2L without annotated orthologs in the *Anopheles gambiae* genome (Figure 1) by the PhyML software [51] used in comparative analysis of drosophilids and vertebrates [58]. While the divergence of vertebrates measured at four-fold degenerate sites of genes encoding ribosomal proteins is very similar to the whole genome estimation [58], the phylogenetic distances for drosophilids are significantly smaller. The phylogenetic distance measured at four-fold degenerate sites of 306 non-conserved genes exceeds the genome average data (Figure 1) and is 1.7–2 times greater than the distances determined for highly expressed ribosomal genes. A similar tendency was observed with pairwise distances estimated using the MEGA4 software [50] but the difference was less pronounced (data not shown). The data suggest that highly expressed genes can introduce a significant bias in estimation of the neutral substitution rate in drosophilids and that the phylogenetic distances reported for drosophilids [58] might be underestimated.

As a comparison we used the divergence of protein sequences for estimation of the relative phylogenetic divergence, calculating pairwise phylogenetic distances of vertebrate and drosophilid species using non-synonymous substitutions in ribosomal genes (Figure 1B). This showing that the relative distances between *D. melanogaster* and other species from the *Drosophila* subgenus (such as *D. virilis*) are similar to or just slightly greater than the distance between human and reptiles, in agreement with results obtained by whole genome analysis [58]. However, it is possible that the highly expressed genes have a lower substitution rate in drosophilids similar to the bias at four-fold degenerate sites (Figure 1A). Our results demonstrate that estimations of phylogenetic divergences in drosophilids and vertebrates vary between different approaches.

### Comparison of UCEs sets between Vertebrate and Fly Species with Similar Phylogenetic Distance

To investigate how the phylogenetic divergence of species affects the number and features of UCEs we identified sequences 100 nt or longer identical in sets of three species of drosophilids or vertebrates (Table 1, Datasets S2 and S3). The use of three species instead of pairwise comparisons reduces the chance of identification of cross-contamination and other errors present in genome assemblies. For flies we used *D. melanogaster*-centric alignments, for vertebrates we used human-centric alignments. *D. erecta* and dog were used as the second species for insect and vertebrate sets, and the phylogenetic difference was associated with divergence of a third species used (Table 1). For convenience the name of the third species was used as the name of the set. As an approximate measure of divergence we used the total length of branches for three species in each set calculated at four-fold degenerate sites in ribosomal genes for vertebrates and in 306 non-conserved Drosophila genes by the PhyML software [51]. As discussed in the previous section these numbers should be regarded as a very approximate estimation.

**Table 1:** UCEs identified in different species.

Species	Distance sbt/site#	UCEs, count	Average length, bp	Median length, bp	UCEs 200+ bp, count	Max length, bp
melanogaster-erecta-						
yakuba	0.348	19,249	124	116	412	520
ananassae	1.322	1,585	117	110	17	301
pseudoobscura	1.445	695	116	110	6	246
virilis	1.915	221	117	110	2	210
grimshawi	1.983	213	119	110	3	210
willistoni	2.024	250	116	110	3	293
mojavensis	2.091	217	119	110	3	293
human-dog-						
cow	0.561	11,016	149	129	1,466	770
mouse	0.745	5,263	142	126	520	770
opossum	1.139	4,040	147	129	518	653
platypus	1.47	2,687	146	129	315	586
chicken	1.504	2,487	148	131	322	610
lizard	1.71	1,287	144	130	138	615
frog	2.068	398	132	122	15	391
zebrafish	2.093	22	119	112.5	0	168
fugu	2.337	20	116	114	1	215

# Distance, in substitutions per four-fold degenerate site, was calculated for ribosomal genes (vertebrates) and 306 non-conserved genes on chromosome 2L (drosophilids).

**Figure 2:**
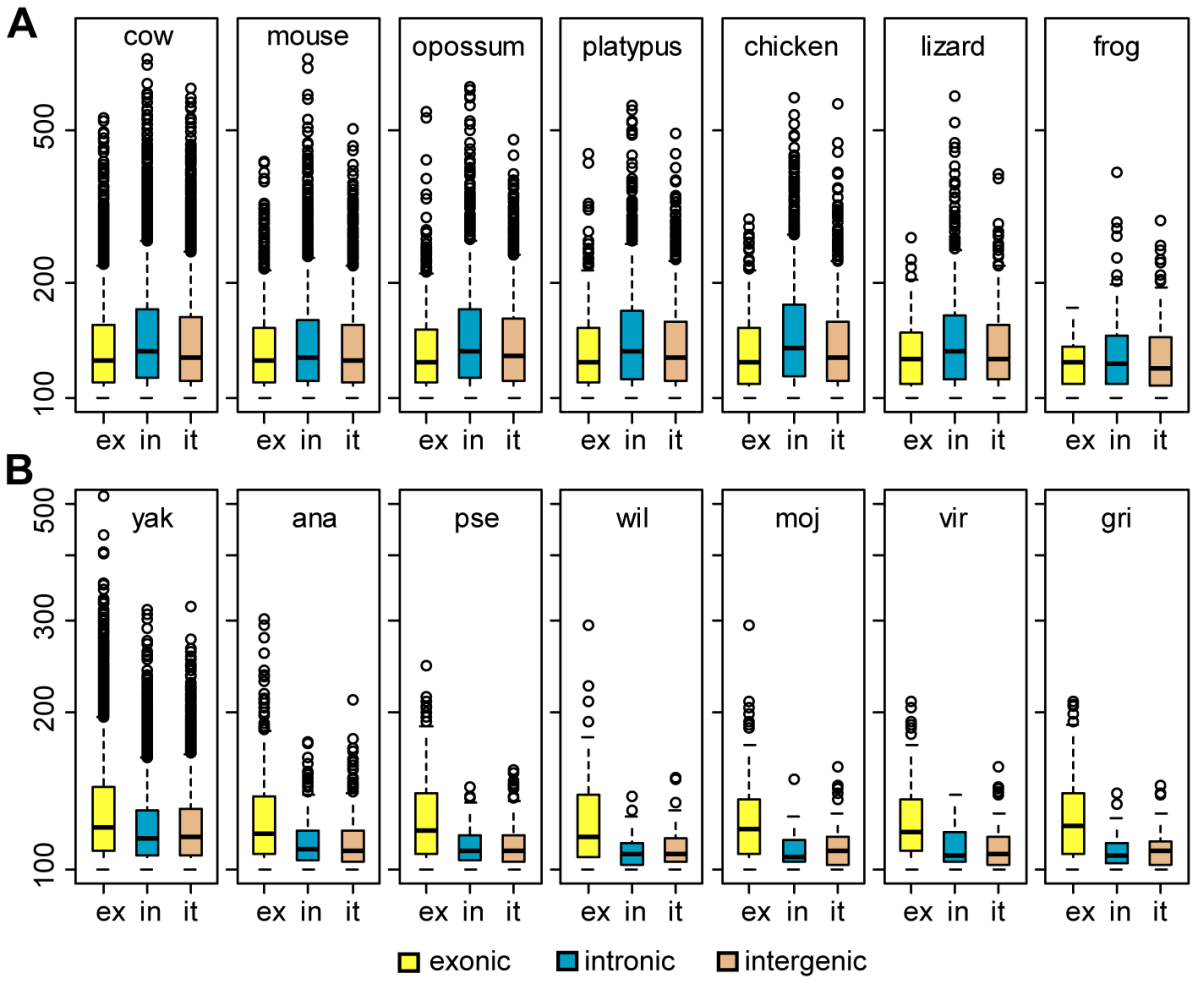
Comparison of UCEs length in vertebrates and flies. (**A**) Vertebrate sets. Data for the UCEs identified in fish are not shown due to a small number of the elements. (**B**) Drosophilids. The abbreviations are the same as on Figure 1. The length of UCEs in bp is shown on the y-axes. The UCEs are split into exonic (ex), intronic (in) and intergenic (it) based on the refSeq genes model. For drosophila the annotation was downloaded on September 16, 2013. Boxes represent upper and lower quartiles, with median shown as a black line. The whiskers were drawn using the R boxplot default 1.5 of interquartile range.

The divergence of the drosophilid sets is either smaller or larger than the divergence of the placental mammals sets (Table 1). However, the divergence in the ananassae and pseudoobscura sets is comparable to the divergence of species in the opossum, platypus and chicken sets, and the remaining drosophila sets are comparable to the lizard and frog sets. Comparison of the sets with similar divergence shows that flies have fewer UCEs than the matching vertebrate species (Table 1), and the elements in flies are shorter (Figure 2). For example, the pseudoobscura set has 695 UCEs compared to 2,687 and 2,487 UCEs in the platypus and chicken sets, respectively. The pseudoobscura set has just 6 UCEs longer or equal to 200 bp, while both platypus and chicken sets have 50 times more elements of the same length (Table 1). In sets with a larger divergence, such as mojavensis and frog, the difference is less pronounced. The number of UCEs declines dramatically in fish sets, apparently due to different selection pressure in this lineage [8]. While the estimations of divergence at four-fold degenerate sites of the mojavensis and zebrafish sets are very close to each other (Table 1), it is worth pointing out that the estimation of relative phylogenetic distances (non-synonymous substitutions) indicates that the fish is more divergent (Figure 1B).

Some sets with similar phylogenetic divergence show a dramatic difference in the number of identified UCEs. For example, the expected number of substitutions per four-fold degenerate site in the ananassae and pseudoobscura sets is 1.3 and 1.4, respectively, but the number of UCEs in these sets differs by more than a factor of two (Table 1). A similar situation is observed in the chicken and lizard sets. We concluded that the number of UCEs does not always reflect the phylogenenetic distance measured by neutral substitutions. We tested whether discordance between a phylogenetic distance and number of observed UCEs could be attributed to a potential difference in the genome assembly quality. We selected 1,136 UCEs from the ananassae set that do not overlap with the UCEs from the pseudoobscura set and mapped these UCEs to the pseudoobscura genome using the liftOver function. Out of 1,136 sequences 1,070 (92%) mapped to pseudoobscura indicating that the orthologous sequences are present in *D. pseudoobscura* for the majority of ananassae UCEs but many sequences do not fit the strict criteria used for UCEs identification due to the presence of substitutions or indels.

The maximal length of UCEs identified in the insect and amniote sets remains more or less the same while the number of UCEs decreases significantly with increasing phylogenetic distance (Table 1). For example, the longest elements identified in the ananassae and mojavensis sets differ in length by just ∼3% despite the fact that the ananassae set contains only species from the *Sophophora* subgenus while the mojavensis set contains species from both the *Sophophora* and *Drosophila* subgenera. The maximal length of UCEs is very similar in all analyzed amniotes sets but declines significantly in the amphibian and fish sets (Table 1). This is in agreement with our previous observation of the elevated substitution rate within eutherian UCEs in the amphibian and fish lineages [8].

We compared UCEs in each set with refSeq gene models in the human and *D. melanogaster* genomes. The majority of UCEs identified in vertebrates and insects are non-exonic, but significant differences exist between the two groups. In drosophilids the proportion of exonic UCEs increases with phylogenetic distance while in tetrapods the fraction of exonic UCEs decreases in distant species (Figure 1). This indicates that in tetrapods the constraints leading to maintenance of the UCEs are stronger in non-coding regions. In all drosophilid sets the density of UCEs in intergenic regions is greater than in introns while the opposite is observed in vertebrates (Table S1). In flies the exonic UCEs are longer than the non-exonic and in mammals the opposite is observed (Figure 2). In fact, the 10 longest UCEs in every fly set analyzed overlap exons of FlyBase genes except for the virilis set in which 9 of the 10 longest UCEs are exonic. In flies the median length of intronic and intergenic UCEs is very similar while in mammals and amniotes intronic UCEs are longer than intergenic. Despite changes in the relative frequencies of exonic, intronic and intergenic UCEs and the general decline in UCE numbers in sets with distant species, the average length of UCEs remains essentially identical in each class of UCEs (Table 1).

### Combined Set of Sophophora UCEs 100+

The criteria used for the identification of UCEs require 100% identity between three distant species. However, UCEs do evolve – albeit at a very slow rate – and such simple criteria would miss some elements. To overcome this problem we identified UCEs in four sets of drosophila species with the phylogenetic distances comparable to those in distant mammals (melanogaster-erecta-ananassae, melanogaster-erecta-pseudoobscura, melanogaster-yakuba-ananassae and melanogaster-yakuba-pseudoobscura) from *Sophophora* subgenus and merged these UCE sets into one superset similar to the approach used for the identification of eutherian UCEs in five placental mammals [8]. The resulting Sophophora UCEs set (Dataset S4) contains 2,126 ultra-conserved elements covering 249.1 kb, with the longest element being just 301 nt long. Almost half of Sophophora UCEs are intergenic (1,018 or 47.9%), 575 (27.0%) are intronic and 533 (25.1%) are exonic (using the refSeq gene models). The combined Sophophora UCEs set has slightly smaller fraction of exonic UCEs than any of the individual UCEs sets suggesting that in flies either exonic UCEs are more conserved or many UCEs avoid detection due to either alignment or assembly problems.

The distribution of Sophophora UCEs varies significantly between chromosomes (Table 2). Only two UCEs were identified on chromosome 4, and the density of UCEs on chromosome X is half of that on chromosome 3R. Regions on long chromosomes adjacent to pericentric heterochromatin are depleted of UCEs (Figure S1). For example, there are no UCEs in the first 2.4 Mb of chromosome 2R assembly or the last 1.6 Mb of chromosome 3L. However, Sophophora UCEs are enriched in regions of intercalary heterochromatin scattered along euchromatin [60]. These regions occupy 14.1% of the *D. melanogaster* genome but contain 450 (21.2%) Sophophora UCEs (1.5 fold enrichment, Chi-square test, *P*-value 1.6E-20). The Sophophora UCEs are enriched in long intergenic regions, at significant distance from annotated genes, *e.g.*, 548 (25.4%) Sophophora UCEs are located at least 5 kb away from any annotated refSeq gene, and such regions occupy 16.2 Mb (13.5%) of the Drosophila genome (1.9 fold enrichment, *P*-value 2.7E-58, Chi-square test). Only 37 Sophophora UCEs overlap known copy number variants, or CNVs [61], 1.5 times less than expected (Chi-square test, *P*-value 0.01).

**Table 2:** Distribution of the Sophophora UCEs in the *D. melanogaster* genome.

Chromosome	Size, Mb	refSeq genes (isoforms)	UCEs, count	UCEs per Mb	UCEs per 100 genes
chrX	22.4	4,208	272	12.1	6.5
chr2L	23.0	4,728	366	15.9	7.7
chr2R	21.1	5195	333	15.7	6.4
chr3L	24.5	4,786	476	19.4	9.9
chr3R	27.9	6,153	677	24.3	11.0
chr4	1.4	291	2	1.5	0.7
Total	120.4	25,361	2,126	17.7	8.4

The vast majority of Sophophora UCEs are conserved in other drosophila species. Even in distant species from the *Drosophila* subgenus (*D. mojavensis*, *D. virilis*, *D. grimshawi*) orthologous sequences were found for about 97% of Sophophora UCEs, with average identity slightly above 91% to the *D. melanogaster* sequence, very similar to the conservation of eutherian UCEs in amniotes [8]. The conservation of Sophophora UCEs declines dramatically outside of drosophilids: only 499, 385 and 429 elements are conserved in mosquito, red flour beetle and honey bee, with an average identity 74%, 69% and 69%, respectively. Among 299 Sophophora UCEs conserved in all three non-drosophilid species, 289 overlap exons of FlyBase protein coding genes indicating that very few non-exonic Sophophora UCEs are conserved in distant insects.

### Sophophora UCEs are Enriched in Conserved Syntenic Blocks but Depleted around Promoters

Many non-exonic UCEs in vertebrates associate with genes involved in regulation of development and transcription [7] and are often embedded within large conserved regions [14], [27]. We analyzed the distribution of Sophophora UCEs in the 22 largest conserved syntenic regions, or homologous collinear blocks, HCBs [52]. Out of 2,126 Sophophora UCEs, 204 (9.6%) overlap with 22 HCBs covering approximately 7.4 Mb (6.1%) of the *D. melanogaster* genome, 1.6 fold more than expected.

**Figure 3:**
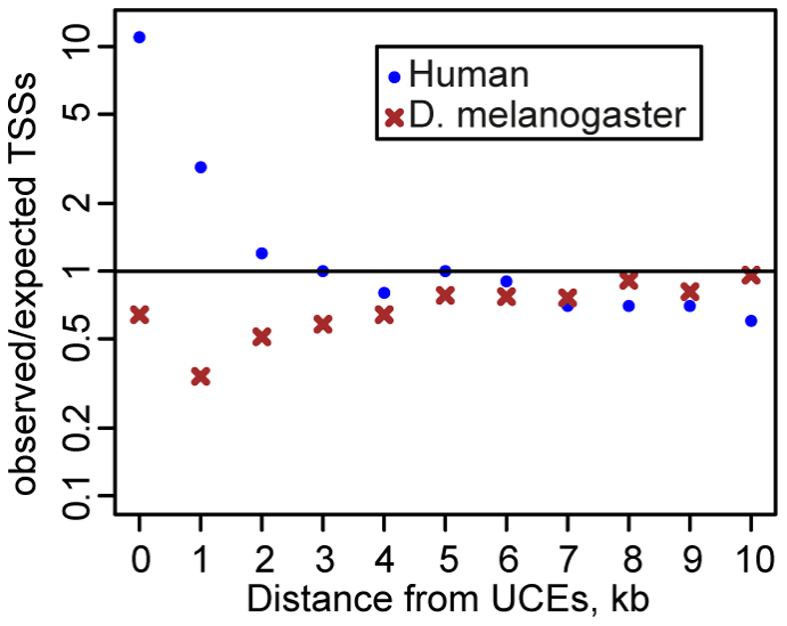
Ratio of the observed and expected refSeq genes transcription start sites in the UCEs and adjacent regions. The eutherian and Sophophora UCEs were used for the human and *D. melanogaster* genomes, respectively. Distance between the UCEs and nearest TSS is on the x-axis. The observed ratio is show on log scale. Expected TSSs were calculated assuming a random distribution.

The transcription start sites (TSSs) of human refSeq genes are enriched in eutherian UCEs and their flanking regions (Figure 3). The enrichment of the eutherian UCEs with the TSSs remains strong even after exclusion of 32 TSSs corresponding to annotated miRNAs. In contrast, in the *D. melanogaster* genome, TSSs of refSeq genes are under-represented within the Sophophora UCEs and flanking regions up to 5 kb (Figure 3). Out of 25 TSSs mapped within the Sophophora UCEs, 12 correspond to 5′ ends of miRNA, snoRNA and snRNAs, and one TSS corresponds to a short non-coding RNA *tre-3* from the *bxd* locus [62]. The TSSs of protein coding FlyBase genes show a threefold decrease in UCEs (data not shown). Within a distance up to 3 kb from the UCEs the number of annotated TSSs is observed to be only half that expected. The Sophophora UCEs are over-represented in gene-poor regions of the intercalary heterochromatin. However, under-representation of TSS near the Sophophora UCEs outside of the intercalary heterochromatin is nearly identical to that observed for the whole genome but with slightly higher *P*-values presumably due to a slightly smaller number of TSSs (Table S2). It seems that in mammals UCEs have somewhat different properties compared to those in flies.

In the human genome 767 refSeq gene TSSs map within 1 kb of the eutherian UCEs. These TSSs belong to 613 genes, 69 miRNAs and 5 snoRNAs. The Gene Ontology annotations [63] were available for 542 genes, and these genes are strongly enriched in the categories linked to development and regulation of transcription (Table S3). Among these genes 164 (30.3%) are assigned to the *transcription factor activity* category (GO:0003700), so 17.2% of 956 genes encoding transcription factors have at least one TSS within 1 kb of eutherian UCEs. Interestingly, among genes not assigned to the GO terms, we noticed four non-coding anti-sense transcripts, DLX6AS, HOXA11AS, OTX2OS1 and SOX2OT, which highlights a putative link between ultra-conservation and regulatory non-coding RNAs [64].

Drosophila genes assigned to categories related to regulation of transcription are also overrepresented among these in proximity to UCEs (Table S3), even though the TSSs of the *D. melanogaster* genes generally do not overlap Sophophora UCEs and flanking regions. Out of 160 protein coding FlyBase genes with TSSs located within or less than 1 kb from the Sophophora UCEs, 122 are assigned to GO categories and 27 (22.1%) are assigned to the category *transcription factor activity* (GO:0003700), corresponding to a 5.9 fold enrichment. However, these 27 genes represent only 7.2% of 374 genes assigned to that category, a significantly smaller proportion than in human.

### Distribution of *P-element*, *piggyBac* and *Minos* Insertions in and around the Sophophora UCEs

We analyzed insertions of transposon-based gene vectors into Sophophora UCEs and neighboring regions in *D. melanogaster*. Out of 52,954 *P-element*, *piggyBac* and *Minos* insertions, 59 are mapped to 52 Sophophora UCEs (Table 3). Both *P-elements* and *piggyBac*s are depleted in regions adjacent to the Sophophora UCEs, and *P-elements* are also depleted from UCEs (Table 4) while the distribution of *Minos* inserts is close to the expected (data not shown). It is well known that transposons are distributed non-uniformly in the genome (Figure S1). Both *P-elements* and *piggyBac*s are under-represented in the intercalary heterochromatin [65], and *P-elements* are strongly biased to TSSs [56] while distribution of the Sophophora UCEs shows the opposite bias. However, the UCEs located outside of the intercalary heterochromatin regions show a low density of insertions in the adjacent regions (Table S4).

**Table 3:** Transposon insertions into the Sophophora UCEs.

Transposon type	Analyzed insertions	in exonic UCEs#	in intronic UCEs#	in intergenic UCEs#	Total	%
*P-element*	33,481	5	7	12	24	0.07
*piggyBac*	15,355	9	9	11	29	0.19
*Minos*	4,118	5	1	0	6	0.15
All inserts	52,954	19	17	23	59	0.11

# As defined by FlyBase Genes 5.12.

**Table 4:** Distribution of the *P-element* and *piggyBac* inserts in the UCEs and flanking regions.

Regions	Size,kb	P-element inserts	Obs/Exp	P-value, Chi test	PBac inserts	Obs/Exp	P-value, Chi test
UCEs	249	24	0.35	5.2E-8	29	0.91	0.6
1 kb	3,880	483	0.45	5.7E-76	260	0.53	7.1E-27
1–2 kb	3,352	536	0.57	1.5E-39	303	0.71	1.0E-9
2–3 kb	3,007	468	0.56	4.4E-38	245	0.64	7.7E-13
3–4 kb	2,748	495	0.65	6.4E-23	237	0.68	8.5E-10
4–5 kb	2,566	450	0.63	1.9E-23	232	0.71	1.0E-7
5–6 kb	2,395	538	0.81	5.5E-7	249	0.82	1.1E-3
6–7 kb	2,251	394	0.63	7.8E-21	216	0.75	2.3E-5
7–8 kb	2,135	432	0.73	2.1E-11	224	0.82	3.1E-3
8–9 kb	2,018	509	0.91	2.6E-2	198	0.77	1.9E-4
9–10 kb	1,927	439	0.82	2.4E-5	216	0.88	5.5E-2

It is possible that the observed low insertion density around the Sophophora UCEs is caused by an inactivation of the marker gene in transposons that may prevent detection of insertions as hypothesized for the intercalary heterochromatin [65]. To test this hypothesis we analyzed the distribution of 2,852 *P-elements* with known suppression status. This set contains 383 insertions with partial suppression of the mini-*white* marker gene [65]. In total, 196 *P-elements* from this dataset are located within 5 kb from the Sophophora UCEs. Among these, 36 are suppressed, which is 1.4 fold more than expected (Chi-square test, *P*-value 0.04). While it does suggest a statistically significant enrichment of suppressed transgenes in proximity to the Sophophora UCEs, the numbers are too small to be definitive.

### Phenotypes Associated with Transposon Insertions into UCEs

Very little is known about the effects of the disruption of non-exonic UCEs in animals. We studied the consequences of transposon integration into Sophophora UCEs. Because it is clear that insertions into genes (exons or introns) could impair gene function due to interference of the insertion with transcription or disruption of the protein-coding region, we focused only on insertions into intergenic UCEs as defined by the FlyBase genes annotation 5.12 (Table 5). Of the 18 intergenic UCEs with insertions, four overlap snRNAs. An insertion in one fly stock, *f02994*, is annotated as an allele of the *Rdl* gene, and it behaves as a recessive lethal [55], [66]. None of the remaining insertions were characterized as lethal or causing sterility in FlyBase. We examined 11 fly stocks carrying insertions in 11 intergenic Sophophora UCEs and identified visible phenotypes in five stocks. In one strain the files have a variegating eye color due to a partial suppression of the mini-*white* marker gene within the transposon integrated into UCE chr2L.116 (Figure S2), three stocks including *f02994* behave as recessive lethals, and a reduced viability was observed in *KG02042* flies (Table 5). However, after rebalancing of the *KG02042* insertion with two different balancers, TM6B-Tb and MKRS, the insertion also behaved as a recessive lethal.

**Table 5:** Transposon insertions into intergenic Sophophora UCEs.

Insertion	UCE position (dm3)	Phenotype
PBac{WH}f06142	chrX:20868423–20868526	None
PBac{PB}c00059	chr2L:7357956–7358059	Variegating eye colour
P{SUPor-P}KG10325	chr2L:12796474–12796574	None
P{EPgy2}snRNA:U2:34ABa^EY07636^	chr2L:13212004–13212116	Not examined
P{EP}snRNA:U2:34ABb^G2309^	chr2L:13215838–13215951	Not examined
P{GSV6}GS15147	chr2L:13244450–13244567	Not examined
P{SUPor-P}snRNA:U2:34ABc^KG07625^	chr2L:13244450–13244567	Not examined
PBac{WH}f02223	chr2L:15466371–15466474	None
P{GSV6}GS14066	chr2R:15626716–15626818	Not examined
PBac{WH}f05912	chr3L:3890591–3890691	None
PBac{WH}Rdl^f02994^	chr3L:9176791–9176898	Lethal
P{SUPor-P}KG02042	chr3L:9294864–9294963	Semi-lethal/lethal
P{EP}EP3253	chr3L:10369759–10369863	Not examined
P{EP}EP2008b	chr3L:10369759–10369863	Not available
PBac{WH}f00315	chr3L:10730347–10730465	Not examined
PBac{RB}e03261	chr3L:10730347–10730465	Not available
P{EP}EP1091	chr3L:10730347–10730465	Not examined
P{XP}d07857	chr3L:12686673–12686786	Lethal
PBac{WH}f07151	chr3L:20943084–20943183	Lethal
PBac{WH}f02632	chr3R:1894272–1894388	None
PBac{PB}c06670	chr3R:9261319–9261447	None
PBac{PB}c06574	chr3R:9261319–9261447	Not examined
P{wHy}snRNA:U1:95Ca^DG12112^	chr3R:19685186–19685357	Not examined

To prove the lethality is caused by the insertions, we performed “phenotype rescue” experiments and removed the transposons from the intergenic UCEs in two fly stocks on chromosome 3L, *d07857* and *KG02042*. Both stocks carry *P-elements* with mini-*white* marker gene responsible for the red eye color in *w* ^−^ genetic background. After excision of the transposon flies would have white eyes. If the lethality is caused by the insertion, we expect to see disappearance of the phenotype in the case of perfect excision of the transposon (“phenotype rescue”), while in the case of partial excision or deletion of DNA adjacent to the integration site we may see a preservation of the phenotype.

**Figure 4:**
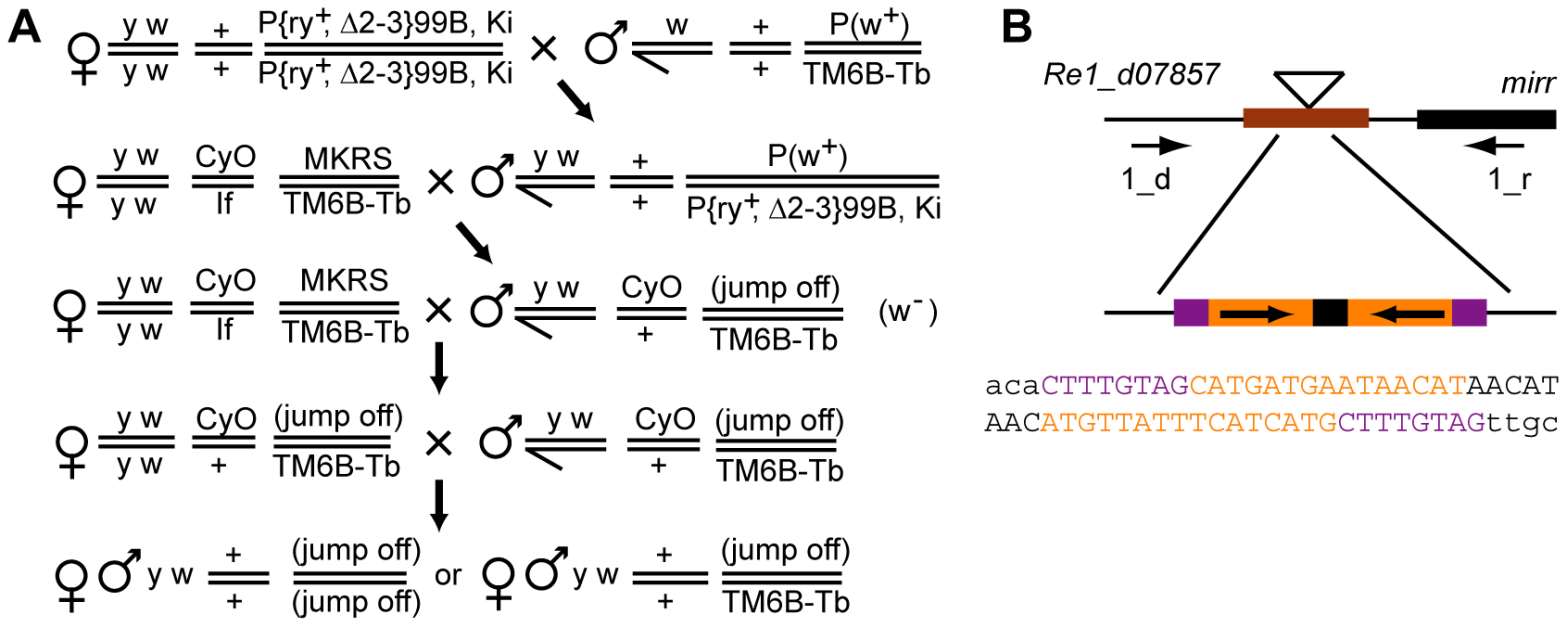
Phenotype rescue experiment. (**A**) Genetic scheme used to remove the transposons from the intergenic UCEs in *d07857* and *KG02042* flies. Males bearing *P-element* insertions were crossed with females carrying the source of transposase on the chromosome 3. In the progeny transposase initiates «jump out» of the *P-element*-based transposons resulting in progeny with white eyes due to absense of mini-*white* marker gene. In the next generation *CyO Tb* males with the white eyes resulted from a complete or partial excision of the transposons were selected and individually crossed with “four balancers” females. The resulted F3 *Tb* progeny were intercrossed and F4 flies were examined on presence of homozigotes with the normal third chromosome. (**B**). Molecular map of the remaining insertion in *Re1-d07857* flies (not to scale). Black bar represent 5′ UTR of *mirr* gene, triangle represent the remaining insertion after the excision of the transposon from the UCE chr3L.296 (brown color). Arrows indicate position of primers used to amplify and sequence the DNA after excision of the transposon. The structure and the sequence of the remaining insertion are shown below. The purple blocks represent 8 bp target site duplication after insertion of the transposon, the orange blocks correspond to remnants of the terminal inverted repeats, and the remaining nucleotides apparently appeared as a results of incomplete excision.

The flies with the inserts were crossed to the flies carrying an active transposase to activate the transgenes (Figure 4), and the flies with white eyes were recovered in the F2 progeny for both *d07857* and *KG02042* stocks indicating that the *P-elements* had jumped out of the original integration sites. Among F4 flies with white eyes we found both restoration of viability (reversion of the phenotype) and maintenance of lethality for both original stocks. We established the *Re1-d07857* fly stock with a complete reversion of the phenotype as a homozygote for the third chromosome and *Re3-d07857* stock with the recessive lethal with *CyO* balancer chromosome and analyzed DNA around the insertion site in these flies.

DNA around the integration site in *Re1-d07857* flies with a complete reversion of the phenotype was amplified by PCR and sequenced. Comparison with the reference sequence revealed an additional 47 bp sequence at the integration site (Figure 4B) suggesting that the presence of the very short insertion in the UCE does not affect the viability. In *Re3-d07857*/*CyO* flies with recessive lethal the PCR produced a single DNA fragment identical to wild type, presumably from the balancer chromosome.

## Discussion

We compared the UCEs in vertebrates and drosophilids and found that both the number and length of UCEs are smaller in insects, in agreement with previous reports [44], [45]. The abundance of UCEs in vertebrates can be partially explained by two rounds of whole genome duplications and the subsequent retention of duplicated copies of the key developmental genes, such as HOX clusters or DLX genes alongside their conserved regulatory elements including UCEs [7]. UCEs can be derived from transposable elements [41] through the process of exaptation, and transposons are abundant in vertebrate genomes. Similar to a recent report [9], the number of UCEs in drosophilid and in vertebrate species negatively correlates with the evolutionary distance between species, however, some sets of species with similar levels of sequence divergence differ significantly in their numbers of UCEs (*e.g.* the ananassae and pseudoobscura sets, Table 1).

A comparison of phylogenetic distances in insects and vertebrates is complicated by differences in their genome structures, such as a shortage of ancient transposons in drosophilids and a strong codon bias in highly expressed Drosophila genes. In addition, methylated CpG sites show a high substitution rate in vertebrates but Drosophila, having lost the CpG methylation system, has a somewhat different mutation pattern. While comparison of divergences between drosophilids and vertebrates is challenging, it is less problematic within each lineage. We also would like to point out that the fly species used in the analysis come from one genus, *Drosophila* (order Diptera, class Insecta), while the vertebrate species include representatives of different classes, *e.g.*, Mammalia, Aves, *etc*. We identified numerous UCEs in species belonging to different classes of vertebrates but none were found between drosophilids and another Diptera species, *Anopheles gambiae* (data not shown). On the evolutionary time scale, the divergence between the most distant drosophila species used in the analysis occurred about 36 million years ago [57] while Eutherian mammals, such as human and cow, diverged over 90 million years ago, according to estimates on the TimeTree [67].

Numerous conserved sequences identical over at least 100 nucleotides are present in sets containing very divergent species (*e.g.*, virilis or frog sets, about two substitutions per neutral site), however, only a few non-exonic UCEs can be traced beyond drosophilids. This suggests that the constraints acting on the non-exonic UCEs were either relaxed outside of drosophilids, or these sequences only came under strong selection pressure in flies after the acquisition of new functions as suggested for amniotes [8].

As in vertebrates, the majority of the UCEs in drosophilds are in non-exonic regions (Figure 1) but significant differences exist between these two groups. In flies the longest UCEs are exonic, and the proportion of exonic UCEs increases in sets with higher phylogenetic distances between member species, whilst in tetrapods the longest UCEs are non-exonic, and the proportion of non-exonic UCEs increases with higher phylogenetic distances between member species. The difference in UCE distribution is also observed between non-coding regions. In contrast to vertebrates, in flies the density of UCEs is lower in introns relative to intergenic regions. The prevalence of exonic UCEs in distant drosophilids might be partially attributed to a strong codon bias for highly expressed genes in drosophila [59]. The abundance of non-exonic UCEs identified in distant vertebrates may indicate a sophisticated regulatory mechanism existing in this lineage.

Sophophora UCEs are depleted near TSSs in the *D. melanogaster* genome whilst the opposite is observed in the human genome. Additionally, in the human genome UCEs are depleted within segmental duplications and copy number variants (CNV), with the strongest depletion observed for non-exonic UCEs [68]. The Sophophora UCEs are under-represented among CNVs but at low statistical significance. In contrast to the human genome, in *D. melanogaster* both exonic and non-exonic UCEs show similar levels of depletion around CNVs: among 37 Sophophora UCEs overlapping CNVs, 10 are exonic – consistent with the proportion of exonic Sophophora UCEs in the whole genome.

Human genes annotated as involved in splicing and RNA binding are enriched with exonic UCEs [7] whilst there is no evidence for a similar enrichment in *D. melanogaster*. The GO analysis of the *D. melanogaster* genes overlapping Sophophora UCEs demonstrates no enrichment for either splicing factors or RNA binding proteins (Table S3) suggesting that this group of genes has no significant contribution to the pool of UCEs in drosophilids. Whilst the GO category ‘Transcription factor activity’ is enriched in genes with exonic Sophophora UCEs, the other enriched categories reference membrane and channel complex proteins which mirrors those of earlier observations [44] and the enrichment is remarkably different from the eutherian UCEs [8].

The Sophophora UCEs are non-uniformly distributed along the *D. melanogaster* genome (Figure S1). The UCEs are depleted in regions adjacent to pericentric heterochromatin but enriched in regions of intercalary heterochromatin with low gene density [60], [69]. These regions are enriched with the Polycomb-group proteins [70], components of the silenced chromatin. In a similar manner, large arrays of highly conserved noncoding elements coincide with Polycomb binding sites and the conserved syntenic blocks in insects [6], [52]. In agreement with this we found an enrichment of the suppressed transgenes integrated in the vicinity of the Sophophora UCEs, as well as a suppression of the marker gene in the *c00059* fly stock carrying the *piggyBac* transposon inserted into an intergenic UCE. While it is tempting to speculate about the involvement of some UCEs in silenced chromatin, the analysis of chromatin data is beyond the scope of this work.

Four out of eleven studied transposon insertions into the intergenic Sophophora UCEs behave as recessive lethals (Table 5) implying important associated functionality in flies. The UCE chr3L.214 associated with lethality in the *KG02042* strain is located within a long intergenic region, more than 13 kb away from the 3' end of the nearest gene, *glutamate receptor IB* (*Glu-RIB*), and more than 30 kb away from the closest promoter of the *PGRP-LA* gene. Removal of a transposon from its integration site restored viability suggesting that the observed lethality is apparently associated with the integration of the transposon. The UCE chr3L.205 associated with lethality in the *f02994* strain is located ∼1.5 kb upstream of the *Rdl* gene. The *piggyBac* insert in this strain is annotated as being an allele of *Rdl* [55], [66]. The two remaining UCEs associated with lethality are located within 1 kb of the transcription start sites of FlyBase annotated genes. The UCE chr3L.296 associated with lethality in the *d07857* fly strain overlaps the promoter region of the current refSeq annotation of the *mirr* gene (checked on March 2013). The UCE chr2L.116 associated with the suppression of the marker gene is located more than 5 kb away from the nearest gene. Our results show that the disruption of UCEs, irrespective of distance from a nearby protein-coding gene, can produce a visible phenotype. Four out of six studied intergenic Sophophora UCEs without any visible phenotype resultant through transposon integration are located more than 5 kb from any nearby gene. For comparison, the mean distance between protein coding FlyBase 5.12 genes in the euchromatic part of the *D. melanogaster* genome is 4.1 kb while the median distance is less than 700 nucleotides.

The “phenotype rescue” experiments performed on two independent fly strains have confirmed that lethality is linked to the disruption of the UCEs by the insertions. Viability was restored by a partial excision of the transposon in the *Re1-d07857* flies, indicating that a small insertion in the same position does not cause lethality. We did not observe a reduction of viability, or any other visible phenotype, in six out of eleven fly strains with intergenic UCEs disrupted by transposons. It is plausible that intact UCEs are only required for survival under natural environmental conditions and hence no visible phenotypes were observed under controlled laboratory conditions. Nonetheless, the high conservation of these sequences still remains a mystery.

## Supporting Information

Figure S1 **Distribution of Sophophora UCEs in the** ***D. melanogaster*** **genome.** The data is shown only for the euchromatic part of the genome. The instances were counted in 100 kb bins. (**A**) Distribution of the UCEs annotated as exonic, intronic or intergenic using protein coding FlyBase gene 5.12 models. Color-coding is shown on each panel. (**B**) Unique transcription start sites of FlyBase genes 5.12. (**C**) *P-element* and (**D**) *piggyBac* insertions with integration sites shorter than 10 nucleotides.
(PDF)

Figure S2 **Partial suppression of mini-** ***white*** **marker gene of the** ***piggyBac*** **transposon inserted into intergenic UCE.** (**A**) Modified screenshot of the UCSC Genome Browser showing DNA around *PBac{PB}c00059* transposon integrated into UCE chr2L.116 (14 kb region, chr2L:7,351,501–7,365,500). Orange marks show integration sites of known transgenes from FlyBase. Black boxes correspond to the Sophophora UCEs, and the refSeq genes are shown in blue. The conservation plot is shown at the bottom. (**B**) Eye color in fly carrying a non-supressed transgene with the mini-*white* gene in *w* ^−^ background. (**C**) Mosaic pigmentation in *c00059* fly. (**D**) Eye of *w* ^−^ fly lacking any pigmentation.
(TIF)

Table S1 **Density of UCEs in introns.**
(DOC)

Table S2 **Distribution of the Sophophora UCEs and TSSs outside of the intercalary heterochromatin regions.**
(DOC)

Table S3 **Gene Ontology analysis.**
(XLS)

Table S4 **Distribution of transposons and Sophophora UCEs outside of the intercalary heterochromatin regions.**
(DOC)

Dataset S1 **List of the refSeq gene IDs used for phylogenetic trees.**
(XLS)

Dataset S2 **BED file with coordinates of UCEs identified in drosophilids.** UCEs identified in different drosophila sets (Table 1). Each BED file contain positions of sequences at least 100 nt long identical in *D. melanogaster*, *D. erecta*, and the third species: yak set, *D. yakuba*; ana set, *D. ananassae*; pse set, *D. pseudoobscura*; wil set, *D. willistoni*; moj set, *D. mojavensis*; vir set, *D. virilis*; gri set, *D. grimshawi*. The coordinates are for the dm3 (BDGP R5) assembly of the *D. melanogaster* genome.
(BED)

Dataset S3 **BED file with coordinates of vertebrates UCEs.** UCEs identified in different vertebrates (Table 1). The coordinates are for the hg19 assembly of the human genome. The vertebrate UCEs were identified in the hg18-centric alignment and the coordinates were liftOvered to the hg19.
(BED)

Dataset S4 **BED file containing Sophophora UCEs for the dm3 genome assembly.**
(BED)
