# Supplementary figures and images for: Comparison of Ultra-Conserved Elements in Drosophilids and Vertebrates
Source: PLoS One. 2013 Dec 13;8(12):e82362. doi: 10.1371/journal.pone.0082362 (PMC3862641; doi:10.1371/journal.pone.0082362)

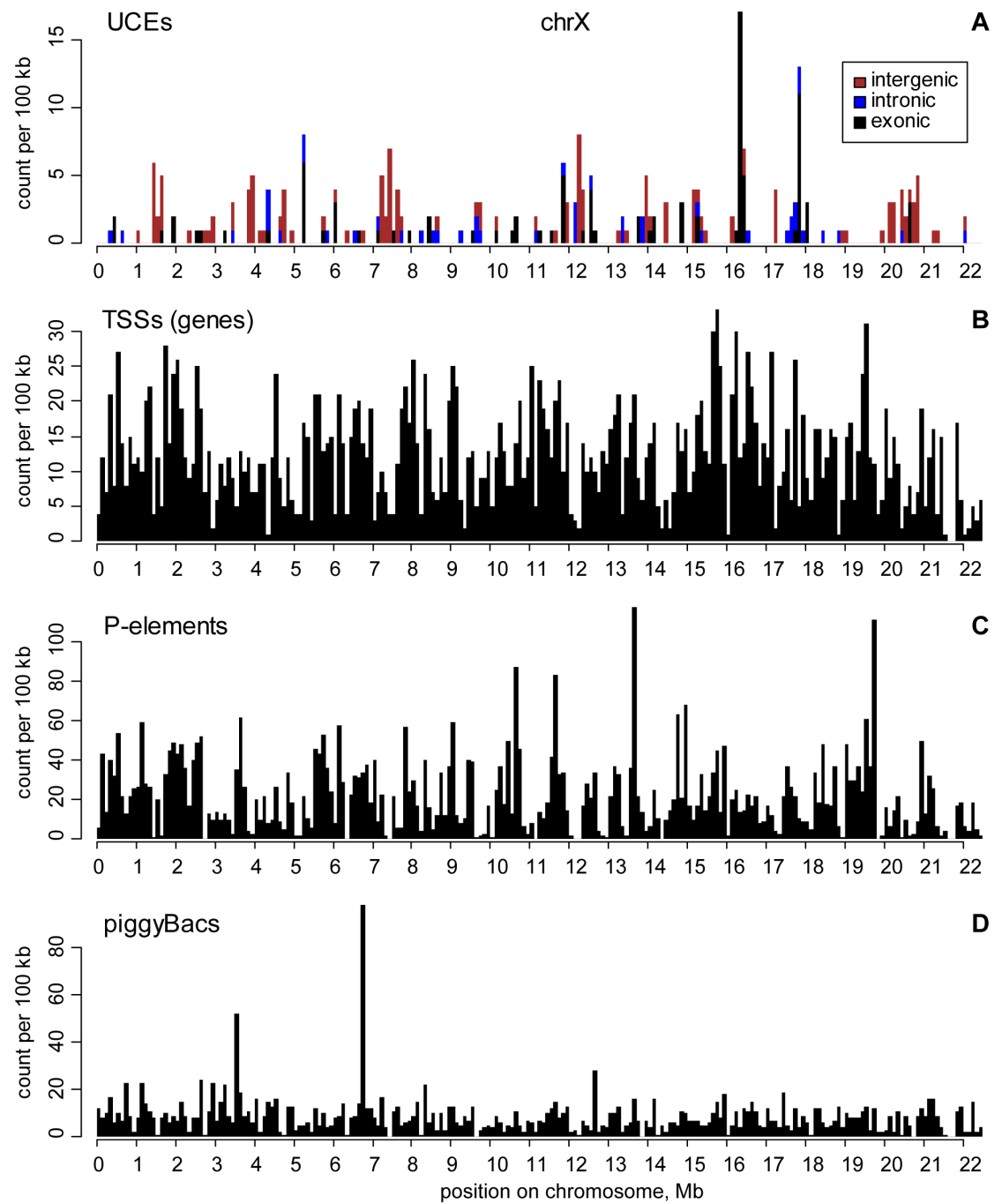

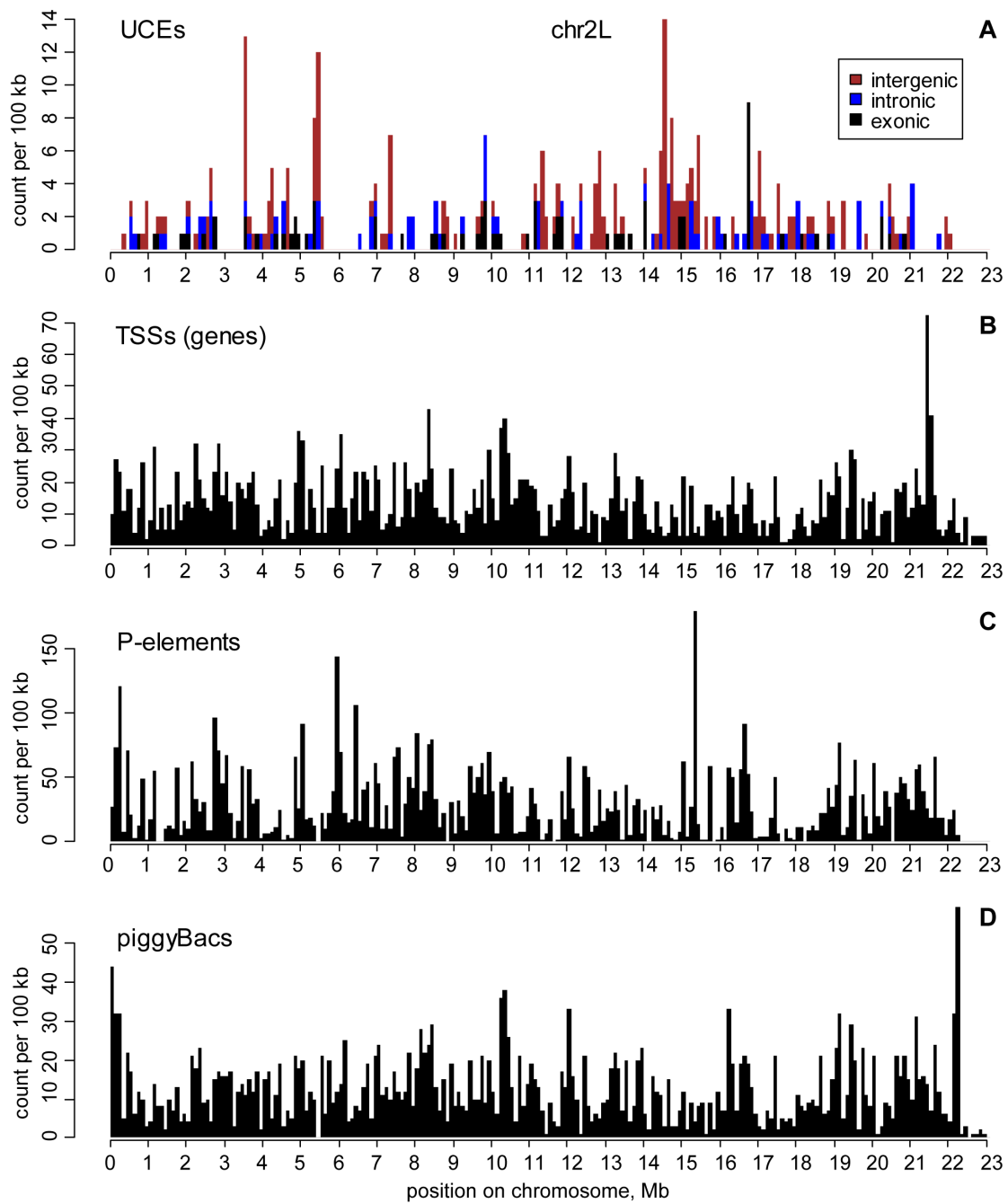

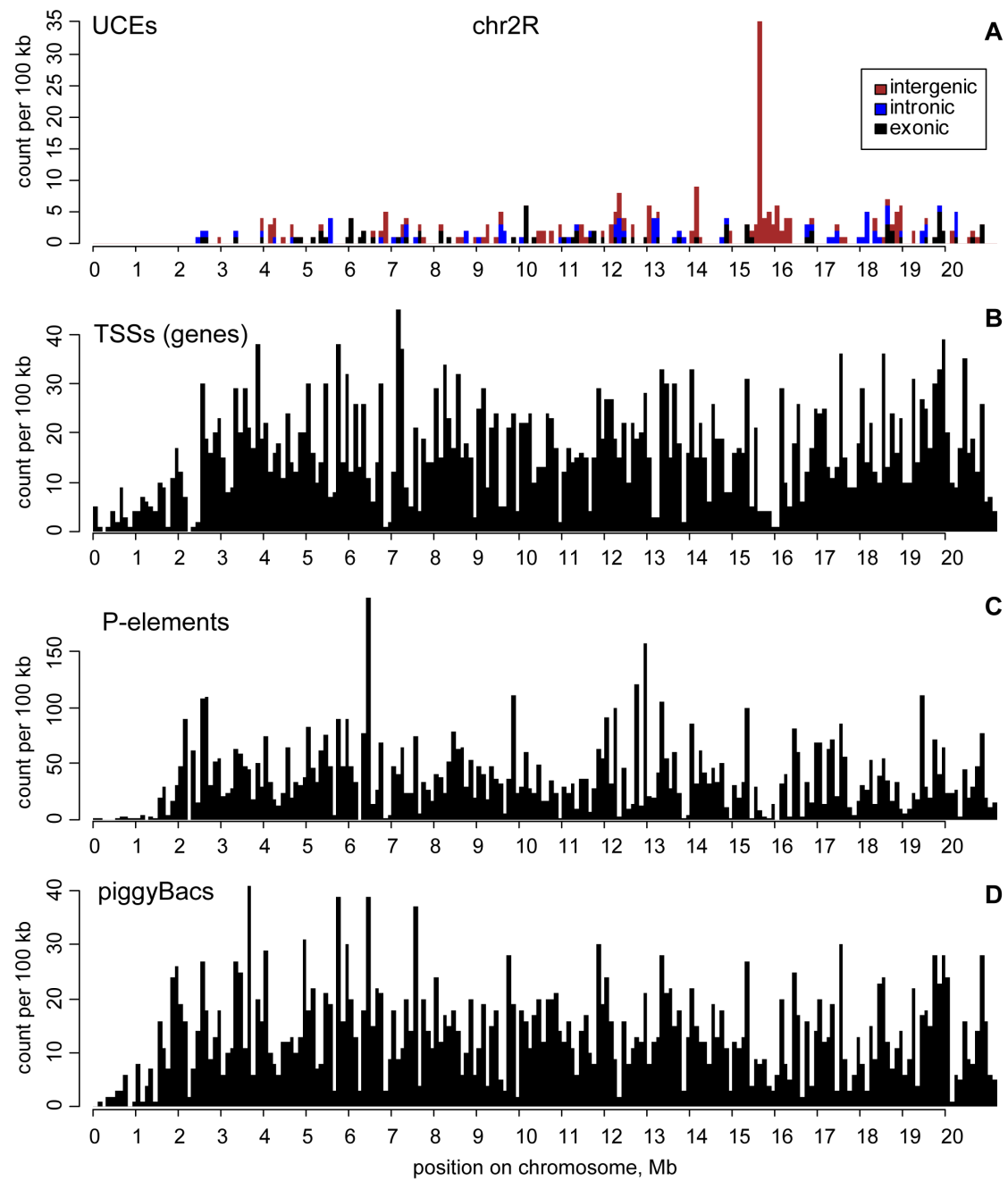

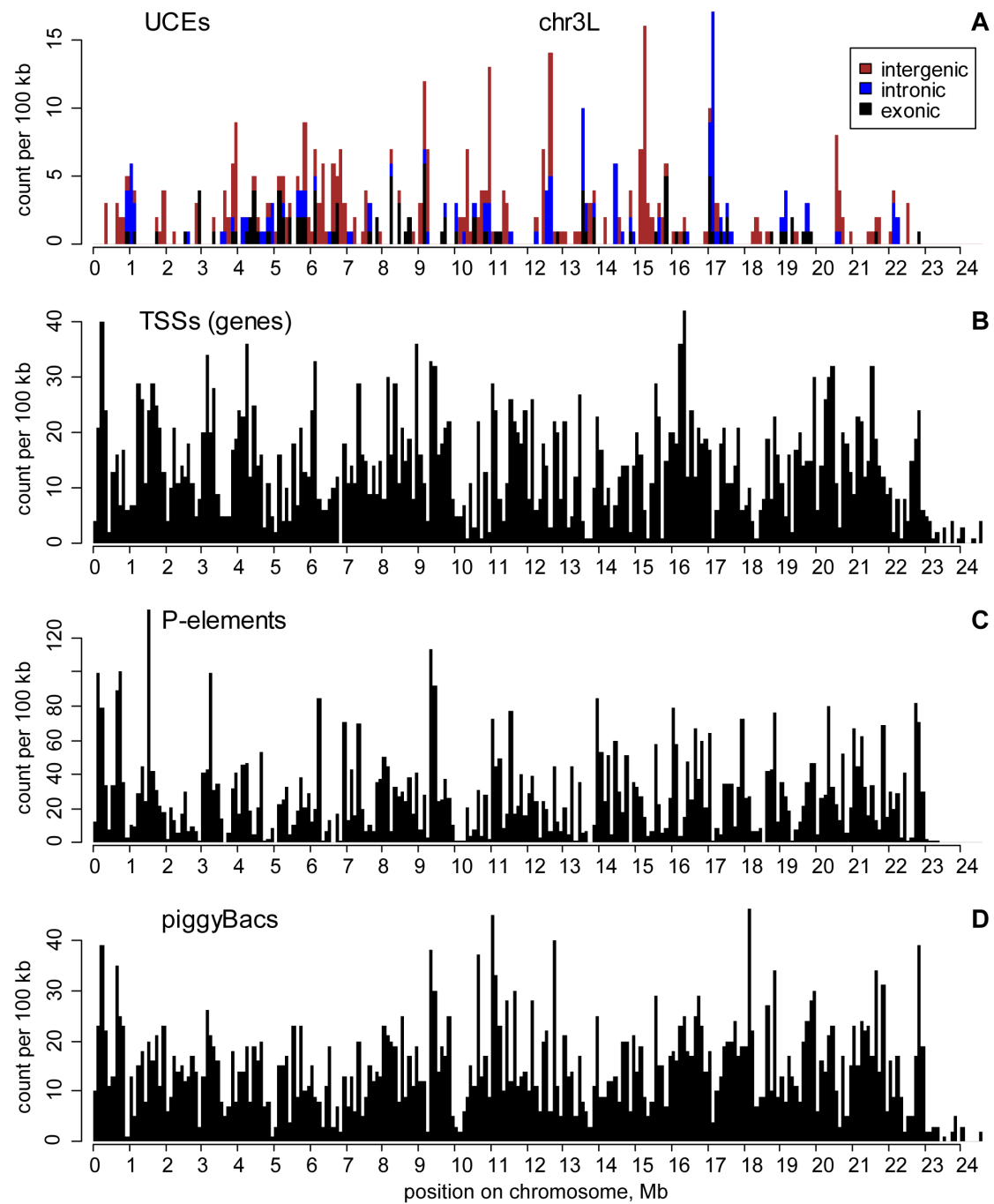

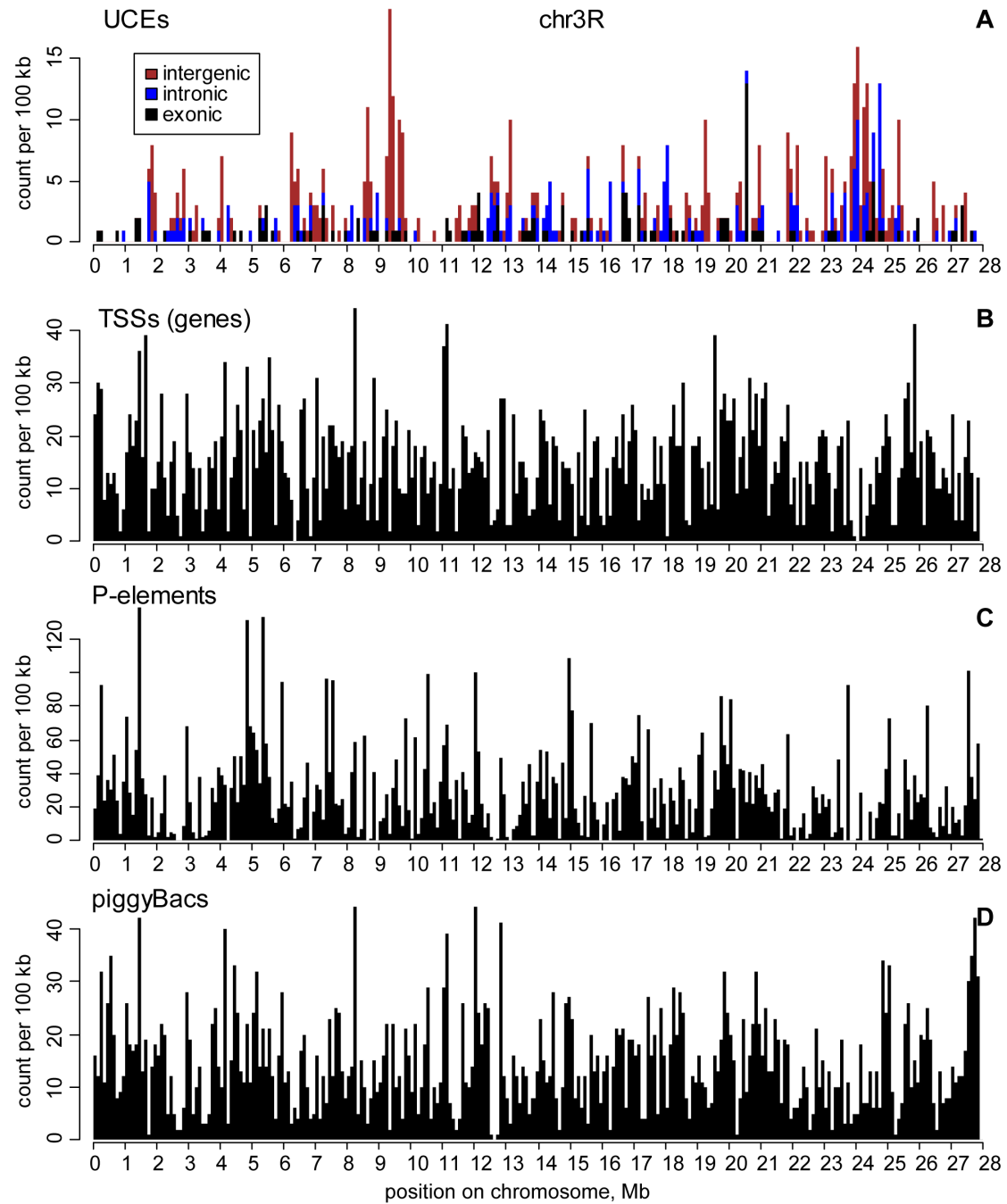

Supplement: Figure S1 — Distribution of Sophophora UCEs in the D. melanogaster genome. The data is shown only for the euchromatic part of the genome. The instances were counted in 100 kb bins. (A) Distribution of the UCEs annotated as exonic, intronic or intergenic using protein coding FlyBase gene 5.12 models. Color-coding is shown on each panel. (B) Unique transcription start sites of FlyBase genes 5.12. (C) P-element and (D) piggyBac insertions with integration sites shorter than 10 nucleotides. (PDF) [file pone.0082362.s001.pdf]

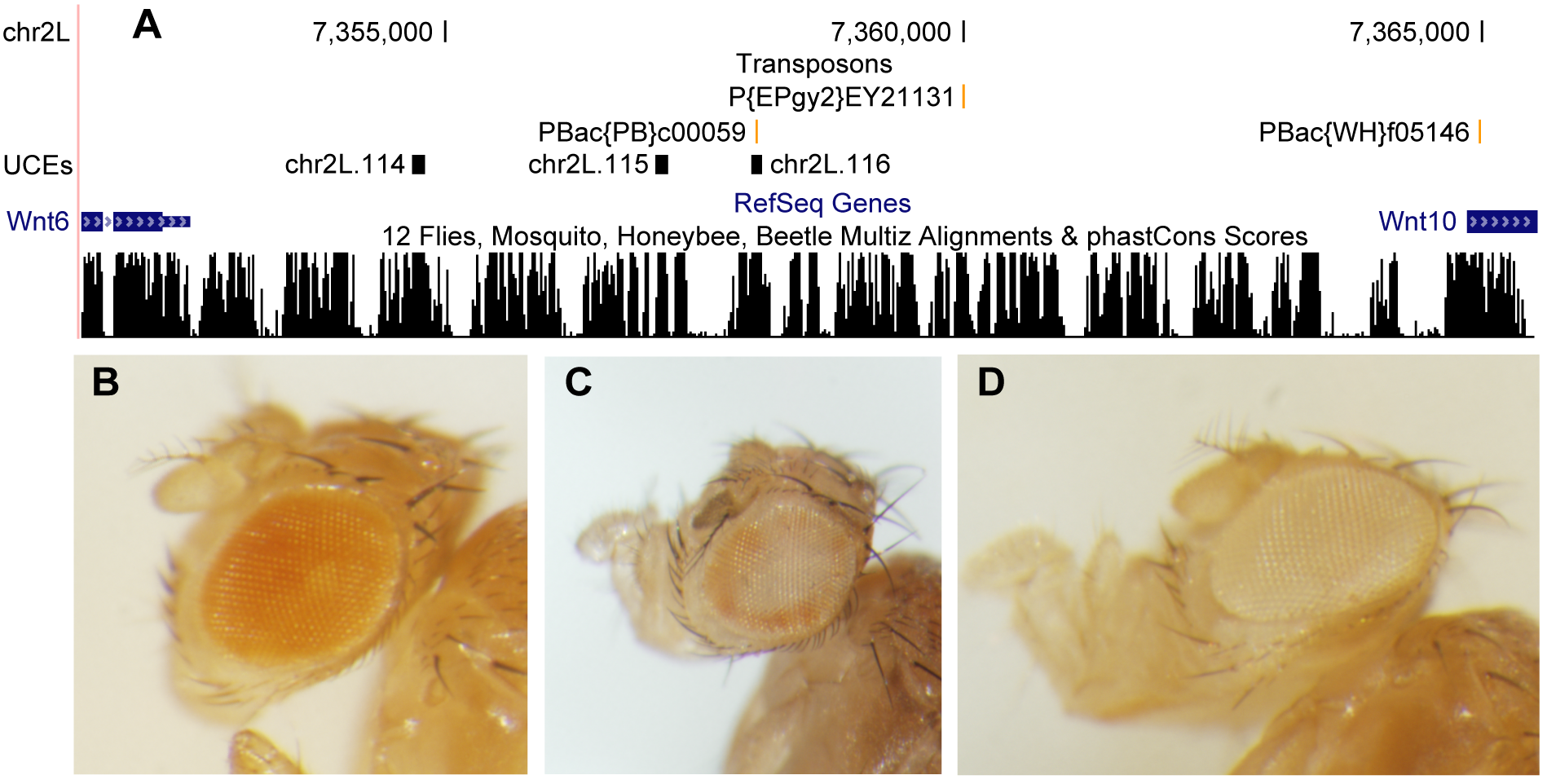

Supplement: Figure S2 — Partial suppression of mini- white marker gene of the piggyBac transposon inserted into intergenic UCE. (A) Modified screenshot of the UCSC Genome Browser showing DNA around PBac{PB}c00059 transposon integrated into UCE chr2L.116 (14 kb region, chr2L:7,351,501–7,365,500). Orange marks show integration sites of known transgenes from FlyBase. Black boxes correspond to the Sophophora UCEs, and the refSeq genes are shown in blue. The conservation plot is shown at the bottom. (B) Eye color in fly carrying a non-supressed transgene with the mini-white gene in w − background. (C) Mosaic pigmentation in c00059 fly. (D) Eye of w − fly lacking any pigmentation. (TIF) [file pone.0082362.s002.tif]
