# Supplementary material for: Comparison of Ultra-Conserved Elements in Drosophilids and Vertebrates
Source: PLoS One. 2013 Dec 13;8(12):e82362. doi: 10.1371/journal.pone.0082362 (PMC3862641; doi:10.1371/journal.pone.0082362)
Supplement: Table S1 — Density of UCEs in introns. (DOC) [file pone.0082362.s003.doc]

Table S1. Density of UCEs in intergenic and intronic regions.

| Set | Intergenic  UCEs / Mb | Intronic  UCEs / Mb | Intergenic / Intronic |
| --- | --- | --- | --- |
| yakuba | 131.2 | 185.0 | 1.4 |
| ananassae | 8.6 | 19.0 | 2.2 |
| pseudoobscura | 3.4 | 8.3 | 2.4 |
| virilis | 0.9 | 2.2 | 2.3 |
| grimshawi | 0.9 | 1.8 | 2.1 |
| willistoni | 1.0 | 2.7 | 2.8 |
| mojavensis | 0.8 | 2.1 | 2.8 |
|  |  |  |  |
| cow | 2.6 | 3.4 | 0.78 |
| mouse | 1.2 | 1.7 | 0.71 |
| opossum | 1.0 | 1.5 | 0.67 |
| platypus | 0.7 | 1.0 | 0.71 |
| chicken | 0.6 | 1.0 | 0.61 |
| lizard | 0.3 | 0.6 | 0.56 |
| frog | 0.1 | 0.2 | 0.67 |
|  |  |  |  |

UCEs were annotated according to refSeq genes (dm3, September 16, 2013; hg19, August 1, 2012).
