# Supplementary material for: Comparison of Ultra-Conserved Elements in Drosophilids and Vertebrates
Source: PLoS One. 2013 Dec 13;8(12):e82362. doi: 10.1371/journal.pone.0082362 (PMC3862641; doi:10.1371/journal.pone.0082362)
Supplement: Table S2 — Distribution of the Sophophora UCEs and TSSs outside of the intercalary heterochromatin regions. (DOC) [file pone.0082362.s004.doc]

Table S2. Distribution of transcription start sites of RefSeq genes in the Sophophora UCEs and flanking regions outside of the under-replicated regions.

| Regions | Size, kb | % of the regions | Observed TSS | Expected TSS | Obs/Exp | P-value, Chi[2] |
| --- | --- | --- | --- | --- | --- | --- |
| UCEs | 198 | 0.2 | 21 | 34.2 | 0.61 | 0.02 |
| 0 - 1 kb | 3043 | 3.1 | 180 | 527.3 | 0.34 | 2.9E-53 |
| 1 - 2 kb | 2611 | 2.6 | 230 | 452.5 | 0.51 | 2.9E-26 |
| 2 - 3 kb | 2342 | 2.4 | 228 | 405.9 | 0.56 | 4.1E-19 |
| 3 - 4 kb | 2126 | 2.1 | 237 | 368.4 | 0.64 | 4.6E-12 |
| 4 - 5 kb | 1979 | 2.0 | 261 | 343.0 | 0.76 | 7.8E-6 |
| 5 - 6 kb | 1845 | 1.9 | 243 | 319.8 | 0.76 | 1.5E-5 |
| 6 - 7 kb | 1737 | 1.7 | 232 | 301.0 | 0.77 | 6.0E-5 |
| 7 - 8 kb | 1643 | 1.7 | 252 | 284.7 | 0.89 | 0.05 |
| 8 - 9 kb | 1567 | 1.6 | 222 | 271.6 | 0.82 | 2.4E-3 |
| 9 - 10 kb | 1507 | 1.5 | 247 | 261.1 | 0.95 | 0.38 |
