# Supplementary material for: Comparison of Ultra-Conserved Elements in Drosophilids and Vertebrates
Source: PLoS One. 2013 Dec 13;8(12):e82362. doi: 10.1371/journal.pone.0082362 (PMC3862641; doi:10.1371/journal.pone.0082362)
Supplement: Table S4 — Distribution of transposons and Sophophora UCEs outside of the intercalary heterochromatin regions. (DOC) [file pone.0082362.s006.doc]

Table S4. Distribution of *P-element* and *piggyBac* insertions in UCEs and flanking regions outside of the intercalary heterochromatin.

| Regions | Observed P-elements | Obs/Exp | P-value, Chi[2] | Observed PBacs | Obs/Exp | P-value, Chi[2] |
| --- | --- | --- | --- | --- | --- | --- |
| UCEs | 23 | 0.39 | 2.1E-6 | 23 | 0.86 | 0.47 |
| 1 kb | 445 | 0.49 | 2.3E-56 | 206 | 0.50 | 7.5E-25 |
| 1 - 2 kb | 512 | 0.65 | 3.0E-23 | 263 | 0.74 | 1.1E-6 |
| 2 - 3 kb | 443 | 0.63 | 1.4E-23 | 210 | 0.66 | 1.2E-9 |
| 3 - 4 kb | 407 | 0.63 | 9.4E-21 | 199 | 0.69 | 1.2E-7 |
| 4 - 5 kb | 420 | 0.70 | 2.3E-13 | 204 | 0.76 | 7.4E-5 |
| 5 - 6 kb | 448 | 0.80 | 3.2E-6 | 203 | 0.81 | 2.6E-3 |
| 6 - 7 kb | 345 | 0.66 | 2.5E-15 | 179 | 0.76 | 2.0E-4 |
| 7 - 8 kb | 373 | 0.75 | 2.2E-8 | 187 | 0.84 | 1.5E-2 |
| 8 - 9 kb | 459 | 0.97 | 0.5 | 173 | 0.81 | 6.2E-3 |
| 9 - 10 kb | 399 | 0.88 | 8.3E-3 | 185 | 0.91 | 0.17 |
